# The true cost of maternal death: individual tragedy impacts family, community and nations

**DOI:** 10.1186/s12978-015-0046-3

**Published:** 2015-06-17

**Authors:** Suellen Miller, José M Belizán

**Affiliations:** Director Safe Motherhood Programs, School of Medicine, Department of Obstetrics, Gynecology, and Reproductive Sciences, University of California, San Francisco, USA; Institute for Clinical Effectiveness and Health Policy, Buenos Aires, Argentina

## Abstract

The death of a woman in pregnancy and childbirth is globally considered an individual tragedy and a human rights violation. Given the inequities in death that occur to marginalized, poor, and vulnerable women in low and middle income countries, there is no doubt that maternal death is a horrific injustice. However, the long term global burden of disease goes far beyond this tragedy. Recent research is demonstrating that there are disastrous consequences in infant and child mortality, loss of economic opportunities, spiraling cycles of poverty in the families and communities where women die giving birth. The journal *Reproductive Health* has published a supplement “The True Cost of Maternal Death,” which includes original research from two major study groups. Harvard’s Francois-Xavier Bagnoud (FXB) Center for Health and Human Rights conducted a multi-country, mixed methods study of the impact of maternal mortality on newborn health and survival, family functioning, interrupted education and economic degradation in four high maternal mortality countries, Tanzania, South Africa, Malawi, and Ethiopia. A collaborative group from Family Care International (FCI), the International Center of Research on Women (ICRW), and the Kenya Medical Research Institute (KEMRI)-Center for Disease Control (CDC)-Research Collaboration conducted research into true costs of maternal death in Kenya. These articles demonstrate the enormous costs that ripple out from the maternal death, and the intergenerational and multi-sectorial disruptions related to maternal mortality. It is important in this period of post-MDG strategy planning period that donors, governments, and NGOs be aware not only of the individual level tragedy of the loss of a mother’s life, but also the financial and health costs associated with maternal mortality, and to keep the focus on maternal health as a key issue in all aspects of development, not just health.

## Editorial

Beginning with the l987 Safe Motherhood Initiative an increased focus has been placed on the significance of maternal mortality as an indicator of the overall well-being of a country’s health indicators, and a high MMR has been seen as linked to gender inequities and overall health system dysfunction. Reduction in maternal mortality was one of the MDG-2015 goals, and, while some progress has been made, there is still a long way to go. Recently the issue of maternal mortality is seen as a proxy for development, women’s empowerment, and economic and social development. While some research has been conducted on higher costs of maternal illness [1], the burden of disease of maternal morbidities (such as fistula and anemia), and the difference in hospital costs between women surviving and women dying [2], little research has looked at the immediate and longer term effects of maternal death on the functioning of the household, the effects on the newborn or other living children, effects on the immediate and extended family members, or on the community.

In this Issue of Reproductive Health, we publish a supplement “The True Cost of Maternal Death,” which includes original research from two major study groups. Harvard’s Francois-Xavier Bagnoud (FXB) Center for Health and Human Rights conducted a multi-country, mixed methods study of the impact of maternal mortality on newborn health and survival, family functioning, interrupted education and economic degradation in four high maternal mortality countries, Tanzania, South Africa, Malawi, and Ethiopia. A collaborative group from Family Care International (FCI), the International Center of Research on Women (ICRW), and the Kenya Medical Research Institute (KEMRI)-Center for Disease Control (CDC)-Research Collaboration conducted research into true costs of maternal death in Kenya. These articles demonstrate the enormous costs that ripple out from the maternal death, and the intergenerational and multi-sectorial disruptions related to maternal mortality. Another striking finding from these studies was the centrality of the woman/mother to all aspects of society, she is truly the center of the home, production, reproduction, social relations, socialization of children, guardians of morality, education and health; if she dies, all functions related to her role suffer. Survivors have to try to cover the variety of activities the mother pursued, but it often takes many people to even partially fill the roles of the one lost mother.

Previous literature from both maternal health and HIV/AIDS research has made clear the link between maternal mortality/morbidity and newborn health; pregnancy and delivery complications affect neonates and increase the risk of stillbirth and neonatal deaths [3,4]. If the infant survives, but the mother does not, nutritional/feeding problems due to lack of breastfeeding, dirty water, or inadequate artificial feeds will often kill the infant or increase risk of infection/stunting [5-7]. Surviving older children often suffer from school dropouts, disrupted education and living arrangements, and, especially for girl children, early marriage, early childbearing, and increased risk of maternal mortality/morbidity [8-11]

The study from Ethiopia [12] explicitly compares the disastrously high rate of neonatal mortality related to maternal mortality. Eighty-one percent of babies of mothers who died also died; babies whose mothers died had a statistically significant 46 times greater risk of dying before one month of age than infants of surviving mothers. But the effects found by this longitudinal study, (using HDSS data and comparing survival trajectories and age specific mortality rates between 1987 and 2011), were long ranging and showed deleterious effects on older children as well. Although older surviving children did not have significantly different probabilities of death, these children were significantly more likely to be unschooled, leaving these children victims to the cycle of poverty, with attendant higher risks of repeating maternal and neonatal mortality. The other study from Ethiopia [13] in the Supplement did show poorer health care for orphaned children with lower rates of immunizations and medical care seeking if ill. This was a qualitative study of the impacts of maternal mortality on living children and families, using in-depth interviews with family members and stakeholders, and focus groups with community members. It was found that fathers rarely assumed child care responsibilities, as the gendered roles of the dual parent family assigns those roles to women, as well as home care, cooking, and developing outside income, such as marketing or farming. Husbands were not only bereft, but ill-prepared to handle the role expansion required after losing a wife. Economic deprivation and poverty often followed maternal death, as not only additional income was lost, but also huge debt was acquired through hospital bills, funeral costs, and time away from paid labor for funeral ceremonies. Some families needed to sell assets; others go into debt borrowing to afford the funeral. Surviving children suffered, with girl children faring worse, often having to leave school to assume maternal roles.

Bazile et. al conducted qualitative in-depth interviews to assess the impacts of maternal death on surviving children, the family and community in rural Malawi [14]. They found that the loss of the mother greatly exacerbated surviving children’s vulnerabilities to illness and malnutrition, shortened and derailed education, and brought about too early labor participation and too young marriage and/or parenthood. If relatives take in the children the deprivations of adding another child in subsistence living conditions can create tensions, health and schooling issues for the natural children and the orphan. If the father takes on a new wife, the children of the dead woman are often treated as second-class citizens, being deprived of food, comfort, health care, and education, which may now only be expended for the new woman’s offspring.

In South Africa, Knight et. al studied the effects of maternal death on surviving children and families using focus group qualitative methods [15]. Findings were similar to those of other studies in the supplement: high financial costs, lost opportunities for education and sexual and social risk. Emotional effects for the older children were especially striking and disturbing, and adolescents, even those who could not remember their mothers, were noted to have a more difficult transition to maturity, particularly older female children. Finally, as seen in some of the other studies, effects on care givers were complex and disturbing.

What marked the South African study as different from the papers from other countries was that the economic costs were somewhat mitigated by governmental social “grants” targeted for orphans. These grants, called the “foster care grant” provided by the South African government for child support were viewed as essential, but not all families were able to access them. Logistical, communications, and policy barriers stopped the flow of funds; one barrier was the presence of a living father in the home. This is indeed shocking, if a family tries to stay intact post the mother’s death, having the father remain with the children, they may be penalized by withholding of support funds. These types of barriers can lead to further household disintegration or force partially intact families to further disintegrate, while expanded access could help mitigate the multiple ill consequences of maternal death.

Also working on data from a region in South Africa with high maternal mortality, and high prevalence of HIV and TB, Houle et. al [16] examined the Agincourt HDSS dataset and used discrete time event history analysis to estimate risk of dying for children whose mothers survived childbirth compared to children whose mothers experienced a maternal death. In addition they examined differences in risk due to mortality etiology. They found a 15 time increased risk of dying among those children whose mothers died vs. those whose mothers survived (RR 15.2; 95 % CI 8.3,27.9), and those younger than one month were at increased risk compared to older children. If the mother had a co-morbidity, such as HIV/AIDs or TB-related maternal mortality, the risk of the child dying was 29 times that of a child whose mother survived. Houle’s paper quantifies the huge magnitude of risk for infants and young children who lose a mother to maternal mortality where maternal health is also compromised by co-morbidities.

The economic household burden of maternal mortality in rural Kenya was studied by Kes et. al using mixed methods, with focus group discussions to illuminate the economic findings [17]. While transport, health care during the pregnancy, and delivery costs were high, it was the funeral costs that were the most monumental. In their study of surviving families, 87 % had to find financial support, 27 % sold assets, and 15 % borrowed from moneylenders. Across 3 wealth groups, low, middle, and high, funerals cost greater than 100 % of annual per capita household expenditures. Kes calls this a “financial shock,” which was then exacerbated by the loss of the woman’s economic activity, the increased time demands of child care, and the loss of potential wage labor and income devoted to child care.

Pande, et al “continuing with a heavy heart”, studies the consequences of maternal death for child health/survival and household functioning in Kenya [18]. The authors found a great “disruption,” in the households where maternal deaths occurred. The authors examined with mixed methods how neonatal and infant survival varied by mother’s survival status, how household dynamics changed after maternal death, and the coping strategies of surviving adult household members. The authors accessed a large surveillance database from the KEMRI/CDC HDSS, reviewed verbal autopsy data, and collected additional data through a quantitative survey of household socio-economic and demographic status, and then, innovatively, a retrospective narrative design within the quantitative survey. After inquiring about tasks normally performed by the deceased woman, for each task she used to perform, the household was queried on who now performed the task. Figures from the article illustrate how many varied tasks one woman would accomplish, and that even multiple people could no longer keep up with that one woman’s tasks in the wake of a maternal death.

This series of articles makes clear that in 2015 and beyond, primary prevention of maternal mortality is a necessary health, human rights, and social issue. But these findings also point to the need for further research on policies and interventions to protect and support family and community integrity to cope with the far-reaching effects of maternal death, if it should happen. All of the articles give suggestions for policies that might work; all make clear that in addition to the valid human rights argument for prevention of maternal death, the large, lingering burden of disease and emotional and economic crisis that follow poor maternal outcomes is a necessary issue for intersectorial development and to keep women’s, maternal, newborn, and child health central in post-2015 agenda setting.

We suggest further that the physical, social, economic, and human rights burden of disease of maternal mortality, multi-generational and far reaching, should spur stakeholders, indeed, all global citizens, to go beyond agendas and goal setting, and to refuse to permit the unnecessary tragedy of preventable maternal deaths.
